# Multi-Objective Optimization Design and Performance Comparison of Magnetorheological Torsional Vibration Absorbers of Different Configurations

**DOI:** 10.3390/ma16083170

**Published:** 2023-04-18

**Authors:** Guisheng Liu, Hongsheng Hu, Qing Ouyang, Feng Zhang

**Affiliations:** 1 School of Mechanical Engineering and Automation, Zhejiang Sci-Tech University, Hangzhou 310018, China; guisheng0901@163.com; 2 College of Information Science and Engineering, Jiaxing University, Jiaxing 314001, China; 3 School of Mechanical Engineering, Nanjing University of Science and Technology, Nanjing 210094, China; 4 Taizhou Jiuju Technology Co., Ltd., Taizhou 318000, China

**Keywords:** magnetorheological (MR) fluid, torsional vibration absorbers, different configurations, multi-objective optimization

## Abstract

The purpose of this study is to provide a convenient optimization design method for magnetorheological torsional vibration absorbers (MR-TVA) suitable for automotive engines, which is a damper matching design method that takes into account the needs of the engine operating conditions. In this study, three kinds of MR-TVA with certain characteristics and applicability are proposed: axial single-coil configuration, axial multi-coil configuration and circumferential configuration. The magnetic circuit model, damping torque model and response time model of MR-TVA are established. Then, under the constraints of weight, size and inertia ratio, according to different torsional vibration conditions, the MR-TVA mass, damping torque and response time are multi-objective optimized in two directions. The optimal configurations of the three configurations are obtained from the intersection of the two optimal solutions, and the performance of the optimized MR-TVA is compared and analyzed. The results show that the axial multi-coil structure has large damping torque and the shortest response time (140 ms), which is suitable for complex working conditions. The damping torque of the axial single coil structure is generally large (207.05 N.m), which is suitable for heavy load conditions. The circumferential structure has a minimum mass (11.03 kg) and is suitable for light load conditions.

## 1. Introduction

Magnetorheological (MR) fluid is one of the earliest and most widely used smart controllable materials among many MR materials. Under the action of an external magnetic field, the rheological properties of this smart material undergo rapid and reversible changes [1,2]. Therefore, MR fluids are widely used in vehicle engineering [3], medical instruments [4], robot motion system and other fields [5,6]. Among them, MR fluid is used more in the development of linear dampers, while there are relatively few studies on rotary MR dampers [7], especially magnetorheological torsional vibration absorbers (MR-TVA).

During the working process of the internal combustion engine, due to irregular changes in in-cylinder pressure and inertial parts with rigid connections always undergo different engine strokes, so when the excitation frequency is close to the crankshaft resonance frequency, they cause the crankshaft to vibrate uncontrollably [8,9]. The vibration of the crankshaft is multi-dimensional, but because the torsional stiffness of the crankshaft is relatively small compared to other directions, torsional vibration generally occurs in the operating range of the crankshaft, which can lead to engine noise, crankshaft fatigue damage or even breakage [10,11]. To avoid damage from torsional vibration, usually, torsional vibration absorbers are installed on the crankshaft. There is also research conducted on the use of torsional vibration for energy harvesting [12,13], and the vibration energy of the system is collected by using a cantilever beam form combined with piezoelectric, electromagnetic [14,15] and capacitive energy harvester. However, since the torsional vibration of the engine crankshaft is several harmonic vibrations in the engine operating range, this requires the torsional vibration energy harvester to have sufficient bandwidth or certain tunable functions. In addition, it is difficult to integrate the energy harvester in the form of a cantilever beam on a high-speed shaft. Therefore, instead of using an energy harvester to collect torsional energy from the crankshaft to achieve a permanent wireless sensor network energy supply, people prefer to install shock absorbers to dampen the torsional vibration of the crankshaft to ensure the life of the crankshaft. With the rapid development of the automobile industry, the higher the engine power, the faster the crankshaft speed, which makes the traditional engine torsional vibration absorbers such as silicone oil torsional vibration absorbers [16], rubber torsional vibration absorbers and other composite damping torsional vibration absorbers unable to satisfy the torsional vibration damping requirements of the engine under complex working conditions [17,18,19,20]. Due to the special rheological effect of MR materials, some scholars have carried out a series of research on MR rotary damper. Hoang et al. proposed a torsional adaptive tunable vibration absorber design based on MR elastomers and then obtained the adaptive tunable vibration absorber structural parameters by establishing a transmission system model and simulation [21,22]. Nguyen et al. established a simplified torsional vibration system and obtained the optimal damping torque of the torsional vibration system; based on this, the MR damper structure was designed [23]. Ehab et al. developed a new type of torsional vibration damper by combining traditional centrifugal pendulum dampers and MR dampers, studied the torsional vibration attenuation of the rotor system under multiple working conditions and verified the advantages of the hybrid torsional vibration damper [24]. Shenoy et al. developed an adaptive tunable vibration absorber based on MR fluids through the established transmission system. The effect of an adaptive tunable vibration absorber on the torsional vibration of the transmission system under different conditions was studied to evaluate the effectiveness of an adaptive tunable vibration absorber in changing the system resonance amplitude and frequency [25]. Gao et al. proposed a novel design of MR elastomer torsional vibration absorber, verified its rationality through simulation and prototype tests and evaluated its vibration reduction effect [26]. Dong et al. developed an MR torsional vibration control system with variable stiffness and damping, and the effectiveness of the torsional vibration control was verified by simulation [27].

Although many scholars have designed the corresponding MR-TVA for some rotor systems and studied and evaluated their performance, to the best of our knowledge, there are currently few studies on MR-TVA applied to torsional vibration control of engine crankshafts. Existing studies have not taken into account the problem that the internal temperature of MR-TVA will rise and its performance will decrease when designing a torsional vibration absorber, nor do they consider the importance of MR-TVA’s response speed to engine torsional vibration damping. It should be known that the temperature rise of the torsional vibration absorber in operation is quite large [28], while the magnetorheological fluid has a high sensitivity to temperature, which greatly reduces its working performance in a high-temperature environment, thereby greatly weakening the vibration damping effect of MR-TVA [29,30]. Additionally, since the heat provided by the coil accounts for a considerable part of the heat generated by the shock absorber, it is necessary to optimize the MR-TVA magnetic circuit structure to achieve the same damping effect at a smaller current to reduce the heat generated by the coil. In addition, as a semi-active torsional vibration absorber, the response time of MR-TVA for crankshaft vibration damping is very important for automotive engines because engine speed changes are more complex and frequent; if the MR-TVA response time is too long when the engine passes the critical speed, MR-TVA will become a useless load. In order to solve the above problems, three MR-TVAs suitable for different working conditions are proposed; a universal MR-TVA magnetic circuit structure optimization and response time optimization method is provided, which takes into account the inertia ratio associated with the crankshaft system; the response time related to the speed of the engine speed rise; and the damping torque of the shock absorber related to the torsional vibration damping effect. Then, the performance and characteristics of the three MR-TVAs are compared. This design approach has certain significance for the wide application of MR-TVA in the torsional vibration reduction in engine crankshaft in the future.

In this paper, the structure of three MR-TVA design schemes is first developed. The finite element model, magnetic circuit model and damping torque model of the three MR-TVA design structures are established, and the fidelity of the model is verified by finite element simulation. By combining the finite element model and the magnetic circuit model, a high-fidelity damping torque model of the MR-TVA is obtained, which can be used for subsequent optimization. Afterward, the structural mass, the damping torque and the response time of the MR-TVA are taken as the optimization targets. Additionally, the magnetic circuit structure parameters and coil parameters are taken as the design variables. The mass of the MR-TVA, the inertial ring rotational inertia and the overall size of MR-TVA are constraints to optimize the structure. According to different torsional vibration conditions using the genetic algorithm to obtain the optimization results, the performance of the optimization results of the three configurations were analyzed and discussed, which provided some design ideas and direction for the subsequent development of MR-TVA.

## 2. Materials and Methods

### 2.1. Materials

**Figure 1:**
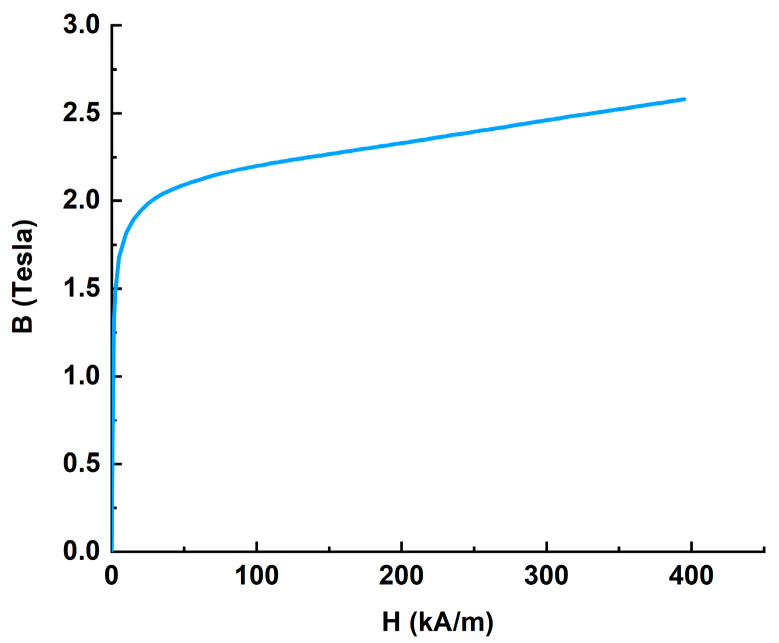
The B–H curve of 1008 steel.

In all configurations, the main structure involved in the magnetic circuit was made of 1008 steel with a density of 7861 kg/m^3^, and the B–H curve of this material is shown in Figure 1.

Figure 1 shows that steel-1008 has a very high magnetic permeability before the magnetic field strength reaches 50 kA/m, but after 50 kA/m it is saturated and the magnetic permeability drops significantly.

**Figure 2:**
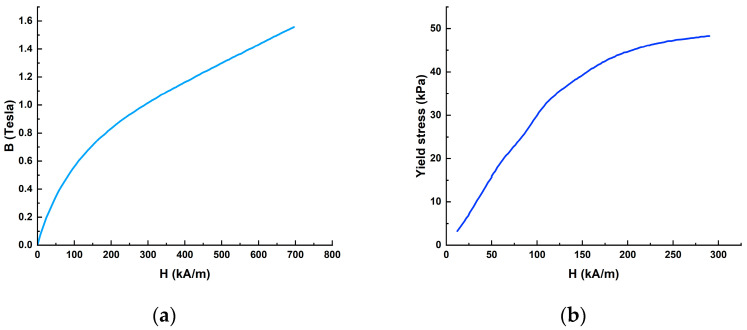
Material properties of MRF-132DG: (**a**) B–H curve; (**b**) the relationship between magnetic field strength and yield stress.

The MR fluid MRF-132DG was developed by Lord Corporation [31], and the material properties are shown in Table 1 and Figure 2.

Figure 2 shows that the magnetic permeability of MRF-132DG is much worse than that of steel-1008, but the permeability of MRF-132DG changes relatively smoothly.

In addition, the electromagnet coil was made of copper, the density was 8900 kg/m^3^, the diameter was 0.8 mm, and the overload current was much higher than the loading current of 1A.

### 2.2. Structure and Magnetic Circuit Design

**Figure 3:**
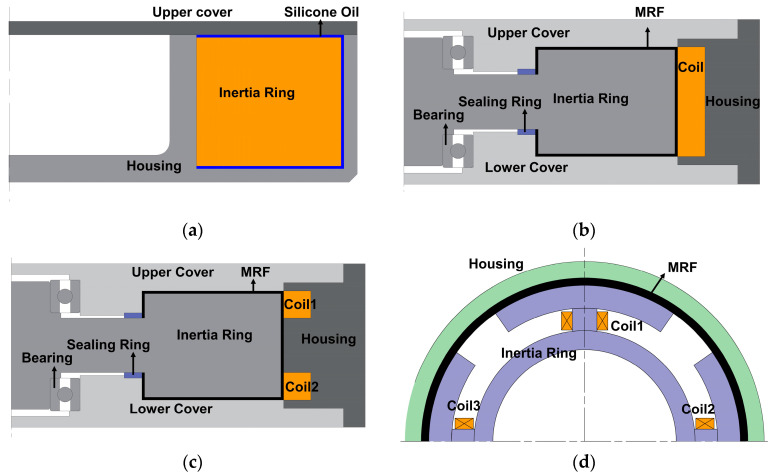
The structure of torsional vibration absorber: (**a**) the structure of the traditional silicone oil torsional vibration absorber; (**b**) axial single-coil configuration; (**c**) axial multi-coil configuration; (**d**) circumferential configuration.

Figure 3a shows the structure of the traditional silicone oil torsional vibration absorber. It works as follows: the absorber housing is flanged to the crankshaft, and the housing is synchronized with the crankshaft at the non-resonant region. The absorber housing drives the absorber inertia ring to rotate in the same direction through fluid friction with the high-viscosity silicone oil. When the crankshaft is in torsional vibration, the rotational speed of the absorber housing fluctuates, and the rotation of the inertia ring lags behind the housing. There is a large rotational speed difference between the housing and the inertia ring, and the energy generated by torsional vibration is finally converted into fluid frictional internal energy to achieve the effect of vibration reduction. The traditional torsional vibration absorber has a narrow effective vibration reduction range, which makes it difficult to apply to projects with increasingly complex working conditions. Based on the rheological properties of MR materials, MR-TVA can achieve adjustable damping force and variable stiffness by changing the magnitude of the external excitation, which makes MR-TVA better adapt to different torsional vibration conditions of the engine, and the system achieves excellent vibration reduction effects throughout the entire operation.

**Figure 4:**
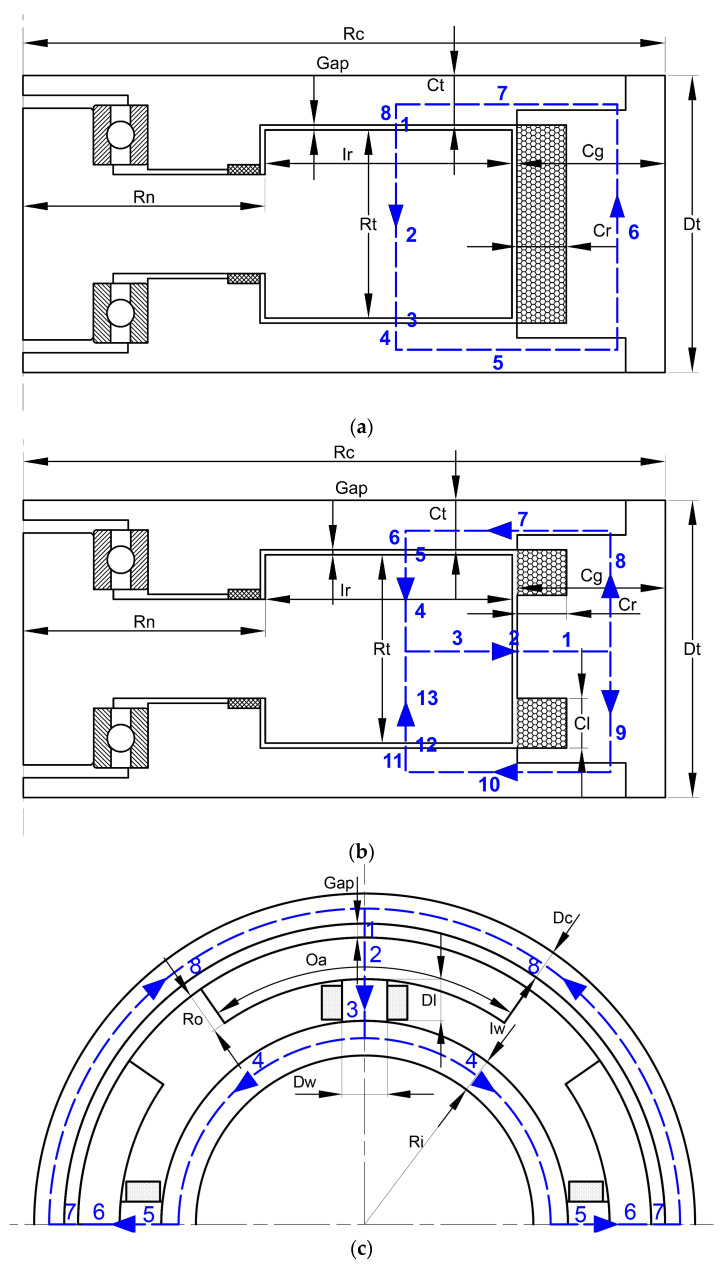
Diagram of the operating principle of MR-TVAs: (**a**) axial single-coil configuration; (**b**) axial multi-coil configuration; (**c**) circumferential configuration.

By taking the traditional torsional vibration damper and rotary MR damper as a reference and considering the magnetic circuit distribution, MR fluid working area and convenient coil mounting position, three MR-TVAs with different characteristics and representativeness were proposed. Additionally, the magnetic circuit distribution of the three MR-TVAs can explain the magnetic circuit distribution of most of the existing rotary magnetorheological dampers and can also provide a certain reference value for the commercialization of MR-TVA for automotive engines in the future. Based on the principle of torsional vibration absorber, three MR-TVAs with different configurations (axial single-coil configuration, axial multi-coil configuration and circumferential configuration) were designed, as shown in Figure 3b–d, which include housing, inertia ring, upper cover, lower cover, excitation coil and MR fluid. It should be noted that the size of the fluid gap is the same in the three configurations; the difference between the axial single-coil configuration and axial multi-coil configuration is the number of coils, and the coils of circumferential configuration are uniformly distributed on the inertial ring. The total number of turns of the coils in the three configurations is equal. When the coils are energized, the MR-TVA generates a closed magnetic field through the housing, cover, MR fluid and inertia ring, which makes the rheological properties change the MR fluid and realize the adjustment of the device damping. The diagram of the operating principle of MR-TVAs is shown in Figure 4.

By taking the external dimensions of an existing engine silicone oil shock absorber as a reference, after magnetic field simulation, it was determined that MR-TVA could meet the working conditions of magnetorheological fluid to give the initial size of MR-TVA. The initial geometric dimensions of MR-TVA are shown in Table 2.

### 2.3. Modeling and Simulation

#### 2.3.1. Magnetic Circuit Model Establishment and Magnetic Field Finite Element Analysis

In order to obtain the damping torque model of MR-TVA, it is necessary to establish a high-fidelity magnetic circuit model, which can also be used for the subsequent optimization of structure and electromagnetic parameters.

Firstly, a two-dimensional axisymmetric finite element model in three configurations was established. The appropriate test point in the fluid gap of the MR fluid in different configurations was selected. Under different ampere turns excitation, they were subjected to magnetic field analysis to verify the constructed magnetic circuit model.

In MR-TVA, the magnetic field is generated by the excitation coil and changes with the applied current. For MR working clearances, the magnetic field can be thought of as the direction of flow perpendicular to the MR fluid. The establishment of the equivalent magnetic circuit is based on the schematic diagram of the magnetic circuit in Figure 4.

Magnetic field analysis is similar to an electric field. When describing the properties of electromagnetic fields, Ampere’s loop theorem and Gauss’s theorem are generally used to characterize:

(1) $$\oint^{\;}\vec{B}\cdot d\vec{l}=\mu_{0}\sum^{\;}I$$

where $\vec{B}$ is the vector of the magnetic field intensity, $d\vec{l}$ is the incremental segment of the closed path L, $\mu_{0}$ is the vacuum permeability, I is the total current enclosed in the closed path, and the sign of I is determined by the integration direction of the closed loop *L* and the right-hand rule.

For the actual magnetic circuit, the magnetic parameters of different magnetic materials are different, so the magnetic properties in the magnetic circuit are constantly changing. It is necessary to simplify the magnetic circuit and write Kirchhoff’s law similar to the magnetic circuit. In a complete magnetic circuit, the medium magnetic flux is a fixed value, ignoring the influence of magnetic flux leakage; Equation (1) can be expressed as:

(2) $$\sum^{\;}H_{n}l_{n}=\sum^{\;}N_{n}I_{n}$$

where $H_{n}$ is the magnetic field in the *n*-th portion along the magnetic circuit with constant magnetic flux. The parameters $l_{n}$ is the length of the portion, $N_{n}$ is the number of turns of the coil, and the $I_{n}$ is the input current to the coil, respectively.

For a magnetic circuit with branches, the inflow and outflow magnetic fluxes are equal at the nodes of the magnetic circuit, which can be expressed as:

(3) $$\sum^{\;}\emptyset_{n}=0$$

According to the hypothetical magnetic circuits of the three configurations of MR-TVA shown in Figure 4, the magnetic circuit can be analogized to a circuit, the simplified magnetic circuit model can be obtained, and a simple magnetic circuit calculation can be expressed as:

(4) $$\sum^{\;}\emptyset_{n}R_{n}=\sum^{\;}N_{n}I_{n}$$

For the magnetic field strength in the fluid gap, it is necessary to calculate the magnetic resistance in the magnetic circuit to obtain the magnetic flux in the magnetic circuit and then obtain the average magnetic field strength of the fluid gap according to the working magnetic circuit area of the fluid gap. The magnetic resistance of the three configurations is obtained from the parameters given in Figure 4 and Table 2.

**Figure 5:**
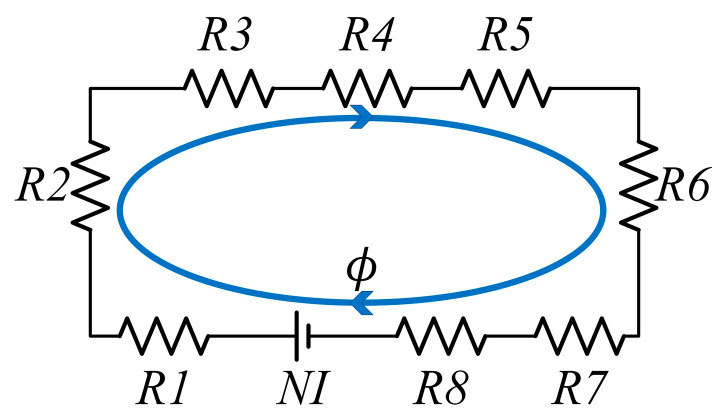
Axial single-coil configuration equivalent magnetic circuit model.

- Axial single-coil configuration;

Axial single-coil configuration magnetic circuit equivalent model and magnetic resistance are shown in Figure 5:

(5) $$\{\begin{array}{l} R_{1,3}=\frac{\mathrm{Gap}}{\mu_{0}\mu_{\mathrm{mr}}{\pi ((R}_{n}{+I}_{r}{)}^{2}{-R}_{n}{}^{2})}\\ R_{2}=\frac{R_{t}}{\mu_{0}\mu_{r}\pi (\left(R_{n}+I_{r}{)}^{2}-R_{n}{}^{2}\right)}\\ R_{4,8}=\frac{C_{t}}{2\mu_{0}\mu_{r}\pi \pi (\left(R_{n}+I_{r}{)}^{2}-R_{n}{}^{2}\right)}\\ R_{5,7}=\frac{\frac{C_{g}+C_{r}+I_{r}}{2}+Gap}{2\mu_{0}\mu_{r}\pi \left(R_{c}-\frac{3C_{g}+C_{r}-2Gap-I_{r}}{4}\right)C_{t}}\\ R_{6}=\frac{D_{t}-C_{t}}{\mu_{0}\mu_{r}\pi [R_{c}^{2}-\left(R_{c}-C_{r}{)}^{2}\right]} \end{array}$$

(6) $$\emptyset_{1}=\frac{NI}{2R_{1,3}+R_{2}+2R_{4,8}+2R_{5,7}+R_{6}}$$

(7) $$B_{P}=\frac{\emptyset }{\pi (\left(R_{n}+I_{r}{)}^{2}-R_{n}{}^{2}\right)}$$

where $\mu_{0}$ is the magnetic permeability of vacuum (4 π × 10^−7^ H/m), $\mu_{r}$ is the relative permeability of the magnetically permeable medium and $\mu_{mr}$ is the relative permeability of the MR fluid; $\emptyset_{1}$ is the magnetic flux of the magnetic circuit; and $B_{P}$ is the intensity of magnetic induction at the midpoint of the fluid gap.

**Figure 6:**
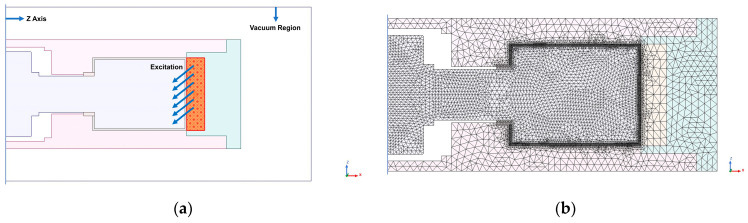
Magnetic field analysis simulation setup of axial single-coil configuration: (**a**) excitation and boundary settings; (**b**) finite element meshing.

**Figure 7:**
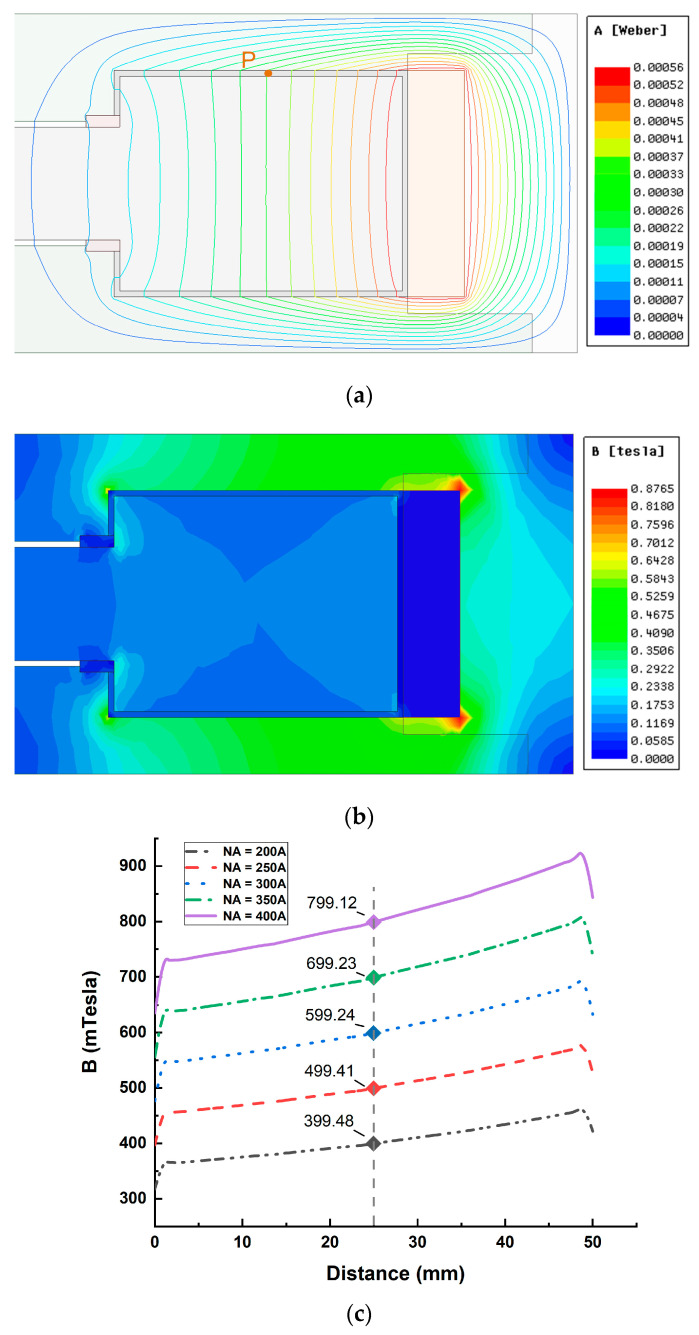
Magnetic field analysis of axial single-coil configuration: (**a**) distribution diagram of magnetic field lines; (**b**) magnetic flux density distribution diagram; (**c**) field strength distribution in the path where point P is located.

A two-dimensional axisymmetric model is established in MAXWELL, and the simulation setup is shown in Figure 6. The magnetic field analysis under different ampere turns excitation is performed on the axial single-coil configuration, and the results are shown in Figure 7.

The curve in Figure 7c shows that the change in magnetic field strength is nearly linear in the radial fluid gap, and the closer to the coil, the greater the magnetic field strength. This is because the closer to the coil, the shorter the magnetic circuit, and the magnetic resistance of the magnetic circuit is smaller. However, the magnetic resistance that varies with the radius occupies a smaller proportion of the magnetic circuit, so the change in magnetic field strength is relatively flat.

Test point P was taken in the fluid gap, as shown in Figure 7a; the simulation and calculation results of the magnetic circuit model are shown in Table 3.

The results in Table 3 show that the difference between the established magnetic circuit model and the finite element simulation model is 4–5%, and the calculation results obtained by the magnetic circuit model are lower than the simulation results. This is because the magnetic field in the fluid gap where the test point is located is exponentially rising (Figure 7c), the position of the test point is not the average point of magnetic field strength, the position of the test point is farther from the coil, and the calculated magnetic resistance is greater, resulting in magnetic field strength of the magnetic circuit model lower than the simulation results.

**Figure 8:**
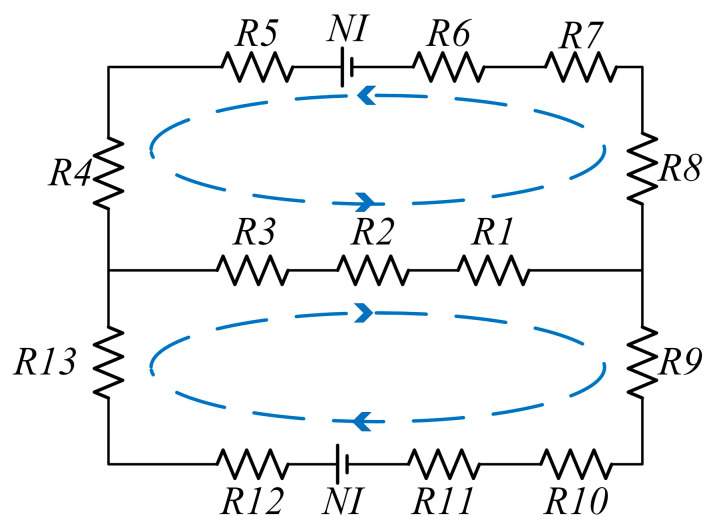
Axial multi-coil configuration equivalent magnetic circuit model.

- Axial multi-coil configuration;

The magnetic circuit equivalent model and magnetic resistance of axial multi-coil configuration are shown in Figure 8:

(8) $$\{\begin{array}{l} R_{1}=\frac{C_{r}+C_{g}}{4\mu_{0}\mu_{r}\pi \left(R_{c}-\frac{3C_{g}}{4}+\frac{C_{r}}{4}\right)\left(D_{t}-2C_{t}-2C_{l}\right)}\\ R_{2}=\frac{Gap}{2\mu_{0}\mu_{mr}\pi \left(R_{c}-C_{g}-\frac{Gap}{2}\right)\left(D_{t}-2C_{t}-2C_{l}\right)}\\ R_{3}=\frac{I_{r}}{4\mu_{0}\mu_{r}\pi \left(R_{c}-C_{g}-Gap-\frac{I_{r}}{4}\right)\left(D_{t}-2C_{t}-2C_{l}\right)}\\ R_{4,13}=\frac{R_{t}}{2\mu_{0}\mu_{r}\pi (\left(R_{n}+I_{r}{)}^{2}-R_{n}{}^{2}\right)}\\ R_{5,12}=\frac{Gap}{\mu_{0}\mu_{mr}\pi (\left(R_{n}+I_{r}{)}^{2}-R_{n}{}^{2}\right)}\\ R_{6,11}=\frac{C_{t}}{2\mu_{0}\mu_{r}\pi (\left(R_{n}+I_{r}{)}^{2}-R_{n}{}^{2}\right)}\\ R_{7,10}=\frac{Gap+\left(I_{r}+C_{r}+C_{g}\right)}{4\mu_{0}\mu_{r}\pi \left[R_{c}-\frac{\left(2Gap+3C_{g}+I_{r}-C_{r}\right)}{4}\right]C_{t}}\\ R_{8,9}=\frac{\left(D_{t}-C_{t}\right)}{2\mu_{0}\mu_{r}\pi [R_{c}^{2}-\left(R_{c}-C_{g}+C_{r}{)}^{2}\right]} \end{array}$$

(9) $$R_{a}=R_{4}+R_{5}+R_{6}+R_{7}+R_{8}$$

(10) $$R_{b}=R_{1}+R_{2}+R_{3}$$

(11) $$R_{z}=R_{a}+\frac{R_{a}R_{b}}{R_{a}+R_{b}}$$

(12) $$\emptyset_{2}=\frac{NI}{R_{z}}$$

(13) $$B_{Q}=\frac{NIR_{a}}{2\pi R_{z}\left(R_{a}+R_{b}\right)\left(R_{c}-C_{g}-\frac{Gap}{2}\right)\left(D_{t}-2C_{t}-2C_{l}\right)}$$

(14) $$B_{P}=\frac{NIR_{a}}{\pi R_{z}\left(R_{a}+R_{b}\right)(\left(R_{n}+I_{r}{)}^{2}-R_{n}{}^{2}\right)}$$

where $\emptyset_{2}$ is the magnetic flux of the magnetic circuit, and $B_{P}$ and $B_{Q}$ are the intensity of magnetic induction at the midpoints of the fluid gap.

**Figure 9:**
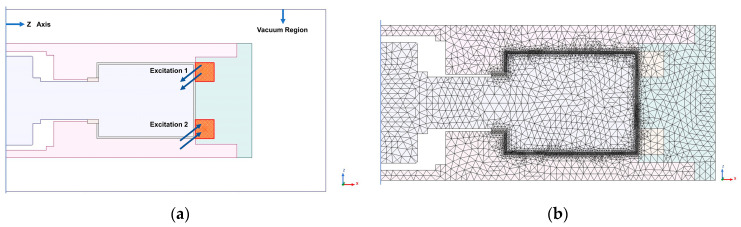
Magnetic field analysis simulation setup of axial multi-coil configuration: (**a**) excitation and boundary settings; (**b**) finite element meshing.

**Figure 10:**
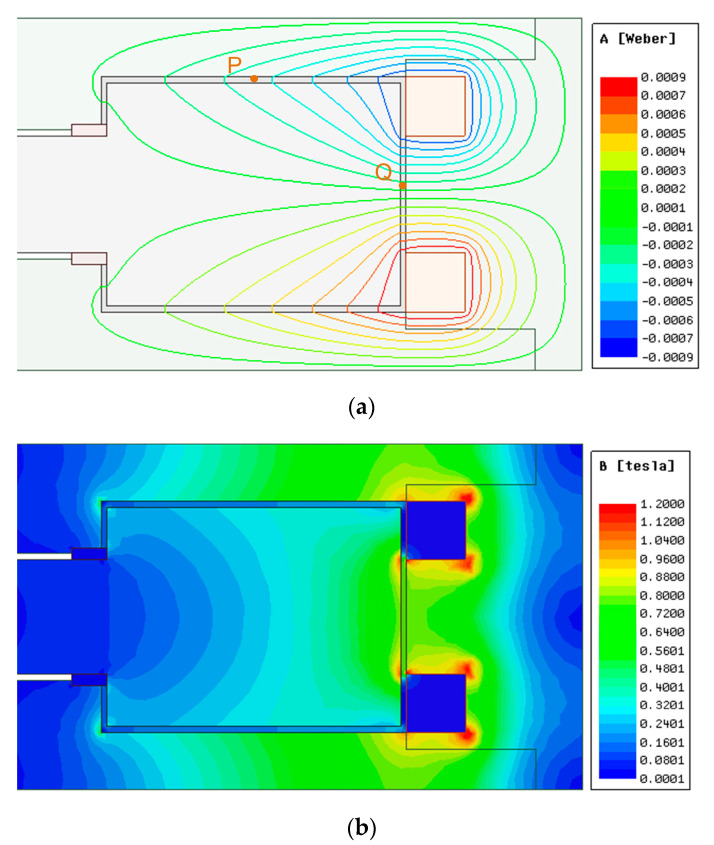

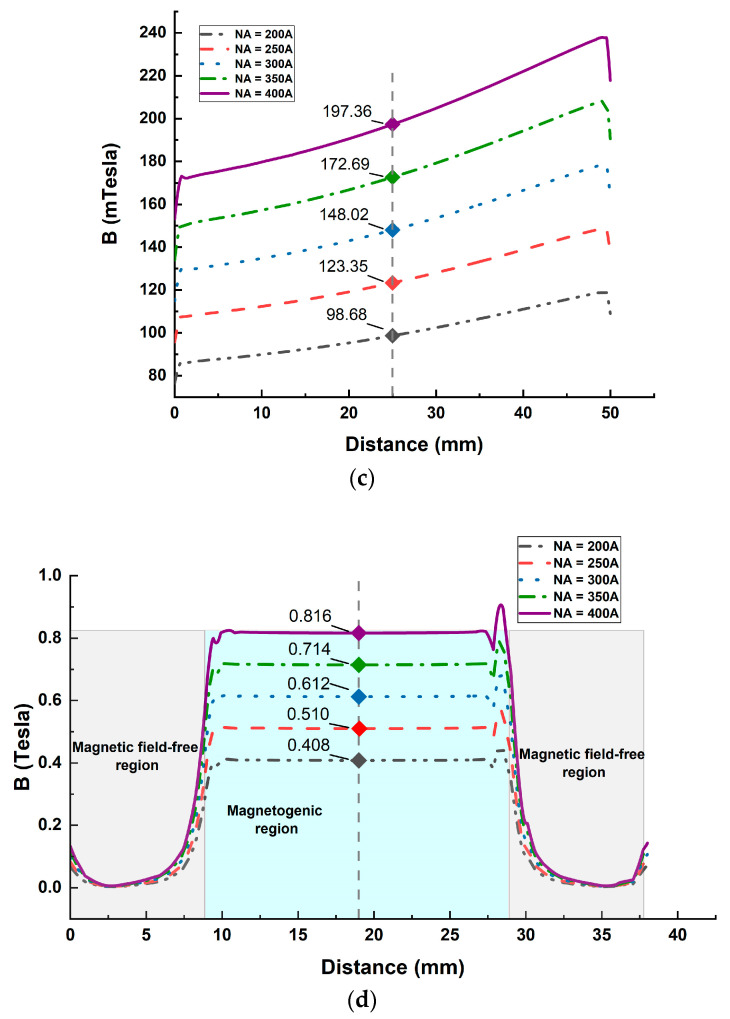
Magnetic field analysis of axial multi-coil configuration: (**a**) distribution diagram of magnetic field lines; (**b**) magnetic flux density distribution diagram; (**c**) magnetic field strength distribution in the path where point P is located; (**d**) magnetic field strength distribution in the path where point Q is located.

The simulation setup of magnetic field finite element analysis of axial multi-coil configuration is shown in Figure 9:

The curve changes in Figure 10c are similar to Figure 7c, but the magnetic field strength in the radial fluid gap is much lower than in Figure 7c. The curves in Figure 10d show that in the circumferential fluid gap, the magnetic field strength in the magnetogenic region is quite high and evenly distributed, but there are fluctuations in the edge region. The magnetic field in the non-magnetic field region on both sides is close to zero, but the magnetic field strength increases sharply in the edge region. This is because the magnetic field generated by the two coils in the radial fluid gap is in opposite directions, while the magnetic field direction is the same in the circumferential fluid gap. Therefore, the magnetic field is weakened in the radial gap and enhanced in the circumferential fluid gap.

Test points P and Q were taken in the fluid gap, as shown in Figure 10a; the simulation results and the calculation results of the magnetic circuit model are shown in Table 4.

The results in Table 4 show that the difference between the established magnetic circuit model and the finite element simulation model is about 4%, and the calculation result of the magnetic circuit model at the P point position is higher than the simulation result, while the calculation result of the magnetic circuit model at the Q point position is lower than the simulation result. This is due to the fact that the magnetic fields generated by the two coils are superimposed at points P and Q, respectively, and the magnetic field generated by one coil will always pass through the fluid gap where point P is located, which is a forward magnetic field. Afterward, it is divided into two magnetic circuits, one through the fluid gap where point Q is located and the other through the fluid gap symmetrical with point P, which is the reverse magnetic field. Then, when calculating the magnetic circuit reluctance through point Q, this part of the magnetic circuit in the magnetic circuit model is more regular (Figure 2b). However, the simulation results show that this part of the magnetic circuit is actually shorter (Figure 10a), so the magnetic circuit resistance calculated in the magnetic circuit model through point Q is higher than the actual one, and the magnetic field strength of the point Q after superposition is lower. The point P situation is similar to point P in the axial single-coil configuration, with the average point of magnetic field strength closer to the coil. The magnetic field intensity at point P calculated by the magnetic circuit model should be lower than the simulation result. However, there are two kinds of forward and reverse magnetic fields at point P, and the magnetic circuit of the reverse magnetic field is symmetrical, so there an extra section of the magnetic circuit is added to the calculation. Therefore, although the magnetic field in both directions of point P in the magnetic circuit model is weakened due to the reverse magnetic field being reduced more, the positive magnetic field of the remaining point P after the superposition of the forward and reverse magnetic fields is higher than the simulation result.

**Figure 11:**
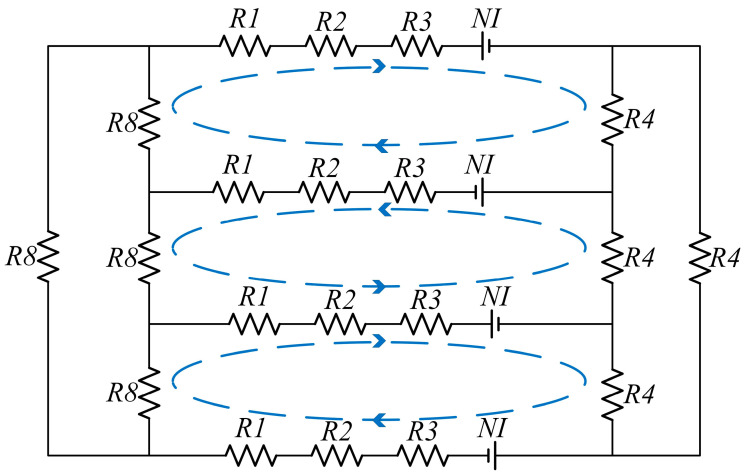
Circumferential configuration equivalent magnetic circuit model.

- Circumferential configuration;

The magnetic circuit equivalent model and magnetic resistance of circumferential configuration are shown in Figure 11:

(15) $$\{\begin{array}{l} R_{1,7}=\frac{2Gap}{\mu_{0}\mu_{mr}\left(R_{i}+I_{w}+D_{l}+R_{o}+\frac{Gap}{2}\right)D_{i}O_{a}}\\ R_{2,6}=\frac{2R_{o}}{\mu_{0}\mu_{r}\left(R_{i}+I_{w}+D_{l}+\frac{R_{o}}{2}\right)D_{i}O_{a}}\\ R_{3,5}=\frac{4D_{l}}{\mu_{0}\mu_{r}\pi D_{w}^{2}}\\ R_{4}=\frac{\pi \left(R_{i}+\frac{I_{w}}{2}\right)}{2\mu_{0}\mu_{r}I_{w}D_{i}}\\ R_{8}=\frac{\pi \left(R_{i}+I_{w}+D_{l}+R_{o}+\frac{D_{w}}{2}\right)}{2\mu_{0}\mu_{r}D_{w}D_{i}} \end{array}$$

(16) $$\emptyset_{3}=\frac{2NI}{2\left(R_{1,7,12}+R_{2,6,11}+R_{3,5,10}\right)+\frac{\left(R_{4,9}+R_{8,13}\right)}{2}}$$

(17) $$B_{P}=\frac{180\emptyset_{3}}{\pi \left(R_{i}+I_{w}+D_{l}+R_{o}\right)D_{i}O_{a}}$$

where $\emptyset_{3}$ is the magnetic flux of the magnetic circuit, and $B_{P}$ is the intensity of magnetic induction at the midpoints of the fluid gap.

**Figure 12:**
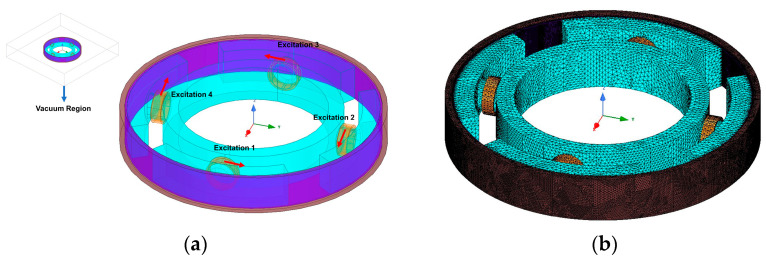
Magnetic field analysis simulation setup of circumferential configuration: (**a**) excitation and boundary settings; (**b**) finite element meshing.

**Figure 13:**
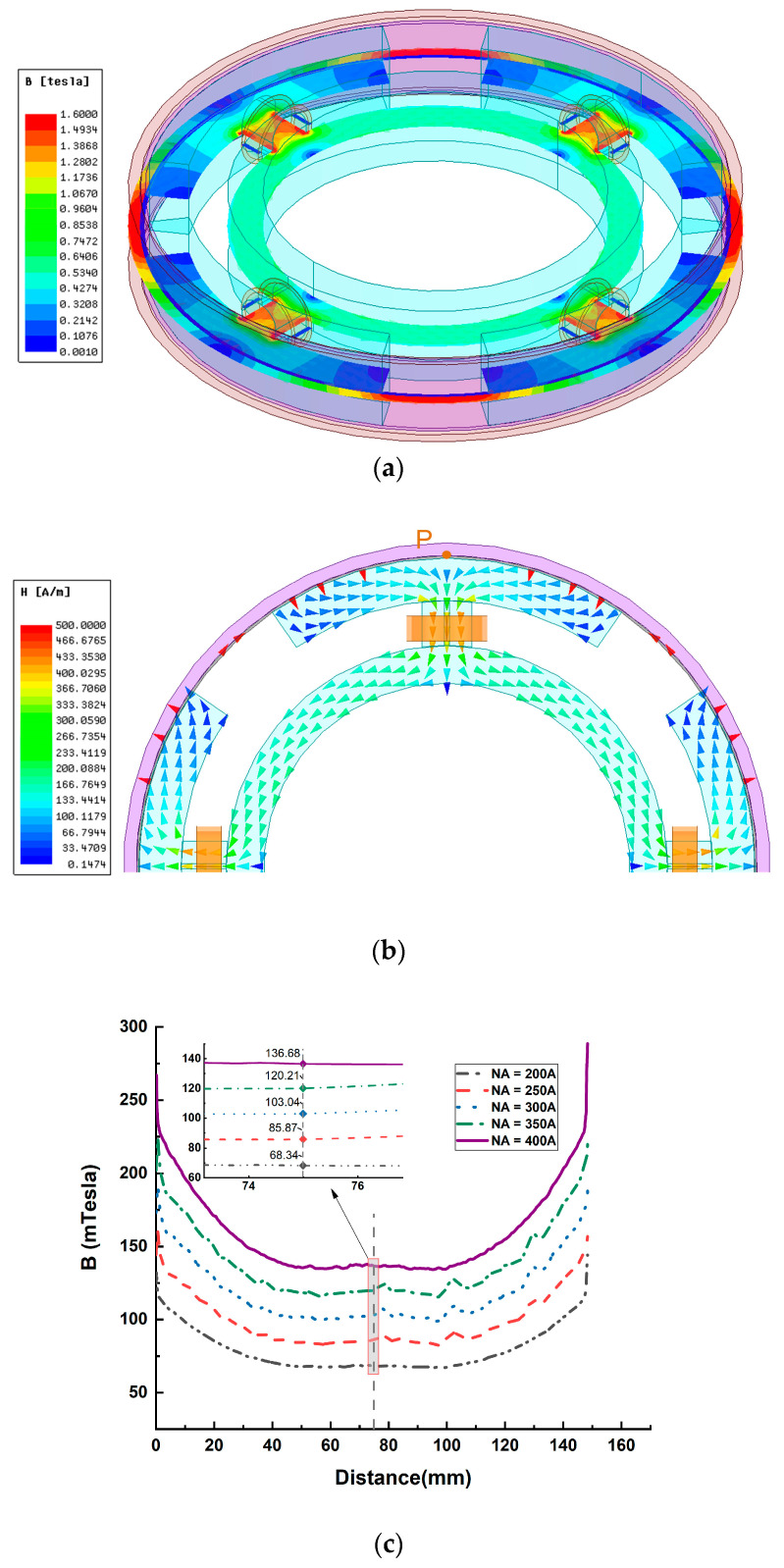
Magnetic field analysis of circumferential configuration: (**a**) distribution diagram of magnetic field; (**b**) distribution diagram of magnetic flux density; (**c**) magnetic field strength distribution in the path where point P is located.

The model was established in the finite element software, the simulation setup of circumferential configuration is shown in Figure 12, magnetic field finite element analysis of circumferential configuration as follows:

The curves in Figure 13c show that in the circumferential gap, the magnetic field strength in the middle of the gap is average, but the magnetic field strength at the edge of the fluid gap will increase. This is because the closer to the edge of the fluid gap, the magnetic circuit will be shorter and the magnetic resistance of the magnetic circuit will be smaller, and the length of the magnetic circuit has a large influence on the magnetic resistance of the magnetic circuit, so the curves in Figure 13c changes to nonlinearity.

Test point P was taken in the fluid gap, as shown in Figure 13a; the simulation results and the calculation results of the magnetic circuit model are shown in Table 5.

The results in Table 5 show that the error between the magnetic circuit model and the finite element simulation model is large, reaching about 8%. This is because when calculating the magnetic resistance of the magnetic ring in the magnetic circuit model, in order to apply to MR-TVA of the same type and different sizes, the whole magnetic permeable ring is considered (Figure 1c). However, the simulation results show that in a closed magnetic circuit, the average line of magnetic field strength does not pass through the entire magnetic permeable ring (Figure 13b), which results in the magnetic circuit model adding four more unnecessary magnetic resistance than the finite element model. Additionally, because the cross-sectional area of the magnetic permeable ring is small, the magnetic resistance of the magnetic permeable ring accounts for a large proportion of the closed magnetic circuit. As a result, the results of the magnetic circuit model calculation are much lower than the simulation results.

#### 2.3.2. Damping Torque Model Establishment

According to the Bingham model of MR fluid, the damping torque $T_{D}$ is mainly composed of two parts; one is the damping torque $T_{\tau }$ generated by the yield stress before and after the rheological effect of the MR fluid in MR-TVA. The other part is provided by the damping torque $T_{\eta }$ generated by the viscosity of the MR fluid. Therefore, the output torque of the MR-TVA can be expressed as:

(18) $$T_{D}=T_{\tau }+T_{\eta }$$

Based on Newton’s law of inner friction and the Bingham model, the equivalent yield stress generated by MR fluid in the fluid gap can be expressed as:

(19) $$\tau =\frac{T}{A}=\tau_{B}+\eta \dot{\gamma }=\tau_{B}+\eta \frac{d_{u}}{d_{y}}=\tau_{B}+\eta \frac{\omega_{r}R}{Gap}$$

where η is the fluid viscosity, Gap is the fluid clearance, and $\dot{\gamma }$ is the shear rate.

- Axial single-coil configuration;

The damping torque of axial single-coil configuration can be expressed as:

(20) $$T_{ad}=\frac{4\pi \tau_{B}}{3}\left(R_{a1}^{3}-R_{n}^{3}\right)+\frac{\pi \eta_{d}\omega_{r}}{Gap}\left(R_{a1}^{4}-R_{n}^{4}\right)$$

(21) $$T_{az}=2\pi R_{a1}^{2}R_{t}\left(\tau_{0}+\eta_{0}\frac{\omega_{r}R_{a1}}{Gap}\right)$$

(22) $$T_{a}=T_{ad}+T_{az}$$

where $\tau_{B}$ is the yield stress of the MR fluid after the rheological effect, $R_{a1}$ is the outer radius of the working gap of the MR fluid, $R_{t}$ is the thickness of the inertia ring, $\mu_{d}$ is the yield stress of the MR fluid after yielding, $\omega_{r}$ is the relative angular velocity between the inertia ring and the housing, $\tau_{0}$ is the zero-field yield stress of the MR fluid, and $\eta_{0}$ is the zero-field viscosity of the MR fluid; other parameters are shown in Table 2.

- Axial multi-coil configuration;

The damping torque of axial multi-coil configuration can be expressed as:

(23) $$T_{bd}=\frac{4\pi \tau_{B}}{3}\left(R_{b1}^{3}-R_{n}^{3}\right)+\frac{\pi \eta_{d}\omega_{r}}{Gap}\left(R_{b1}^{4}-R_{n}^{4}\right)$$

(24) $$T_{bz1}=2\pi R_{b1}^{2}C_{1}\left(\tau_{0}+\eta_{0}\frac{\omega_{r}R_{b1}}{Gap}\right)$$

(25) $$T_{bz2}=\frac{2\pi \eta_{d}\omega_{r}C_{2}}{Gap}R_{b1}^{3}+2\pi \tau_{B}C_{2}R_{b1}^{2}$$

(26) $$T_{b}=T_{bd}+T_{bz1}+T_{bz2}$$

(27) $$R_{b1}=R_{c}-C_{g}-Gap$$

(28) $$C_{1}=2\left(C_{l}-Gap\right)$$

(29) $$C_{2}=D_{t}-2\left(C_{t}+C_{l}\right)$$

where $R_{b1}$ is the outer radius of the working gap of the MR fluid, $C_{1}$ is the working gap width of the MR fluid in the circumferential direction of the zero field, and $C_{2}$ is the working gap width of the circumferential magnetic field of the MR fluid.

- Axial circumferential configuration;

The damping torque of circumferential configuration can be expressed as:

(30) $$T_{cz}=\frac{\pi O_{a}\omega_{r}\eta_{d}D_{i}}{45Gap}R_{c1}^{3}+\frac{\pi o_{a}\tau_{B}D_{i}R_{c1}^{2}}{45}$$

(31) $$T_{cd}=\frac{\pi \omega_{r}\eta_{0}}{Gap}\left(R_{c1}^{4}-R_{c2}^{4}\right)+\frac{4\pi \tau_{0}\left(R_{c1}^{3}-R_{c2}^{3}\right)}{3}$$

(32) $$T_{c}=T_{cz}+T_{cd}$$

(33) $$R_{c1}=R_{i}+I_{w}+D_{l}+R_{o}$$

(34) $$R_{c2}=R_{i}+I_{w}+D_{l}$$

where $R_{c1}$ is the working gap radius of the MR fluid of circumferential configuration, $R_{c2}$ is the inner radius of outer ring of inertia ring.

#### 2.3.3. Response Time Model Establishment

The response time of MR-TVA is mainly composed of coil-related response time and material-related response time, and only the former is discussed in this article. The excitation coil in the shock absorber can be thought of as an RL circuit, and the calculation of the coil response time for the three configurations is as follows.

**Figure 14:**
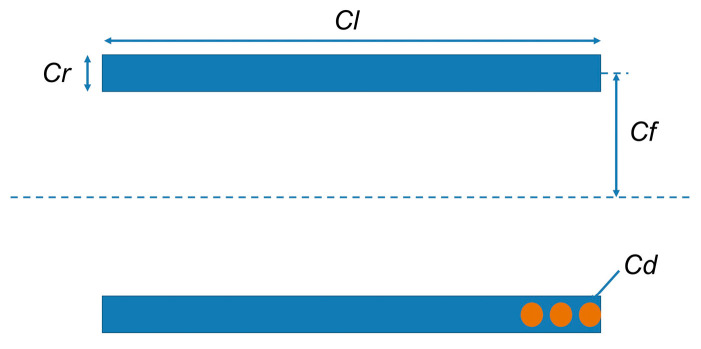
The design variables of coil.

The design variables related to the coil are shown in Figure 14.

The response time of MR-TVA can be expressed as follows:

(35) Z=NA

(36) $$L=\frac{N^{2}\emptyset }{Z}$$

(37) $$R=\frac{\rho l}{A_{c}}=\frac{2\pi C_{f}N\rho }{\pi C_{d}^{2}/4}=\frac{0.168NC_{f}}{C_{d}^{2}}$$

(38) $$L\frac{d}{dt}i\left(t\right)+Ri\left(t\right)=V\left(t\right)$$

(39) $$i\left(t\right)=\frac{V}{R}\left(1-e^{-\frac{R}{L}t}\right)$$

(40) $$t=4\frac{L}{R}$$

where Z is the number of ampere-turns, N is the turns of the coil, A is the current in the coil, L is the inductance of the coil, R is the resistance of the coil, ∅ is the magnetic flux of the magnetic circuit, ρ is the resistivity of the coil (0.021 Ω·mm^2^/m), $C_{d}$ is the diameter of the copper wire, and *t* is the time it takes for the coil’s control current to reach 98% of the target value.

- Axial single-coil configuration;

(41) $$N_{1}=\frac{C_{r}\left(R_{t}+2Gap\right)}{C_{d}^{2}}$$

(42) $$R_{1}=\frac{0.168N_{1}\left(R_{i}+I_{r}+gap+\frac{C_{r}}{2}\right)}{C_{d}^{2}}$$

(43) $$L_{1}=\frac{N_{1}^{2}\emptyset_{1}}{Z}$$

(44) $$t_{1}=\frac{4L_{1}}{R_{1}}$$

where $\emptyset_{1}$ is the magnetic flux of the magnetic circuit of axial single-coil configuration (Equation (6)), $N_{1}$ is the turns of coil of axial single-coil configuration.

- Axial multi-coil configuration;

(45) $$N_{2}=\frac{C_{r}C_{l}}{C_{d}^{2}}$$

(46) $$R_{2}=\frac{0.168N_{2}\left(R_{i}+I_{r}+gap+\frac{C_{r}}{2}\right)}{C_{d}^{2}}$$

(47) $$L_{2}=\frac{2N_{2}^{2}\emptyset_{2}}{Z}$$

(48) $$M_{2}=\frac{L_{2}}{2}$$

(49) $$t_{2}=\frac{4\left(2L_{2}-2M_{2}\right)}{2R_{2}}$$

where $\emptyset_{2}$ is the magnetic flux of the magnetic circuit of axial multi-coil configuration (Equation (12)), and $M_{2}$ is the coil mutual inductance of coil of axial multi-coil configuration.

- Axial circumferential configuration;

(50) $$N_{3}=\frac{C_{r}\left(D_{l}-1\right)}{C_{d}^{2}}$$

(51) $$R_{3}=\frac{0.084N_{3}\left(D_{w}+C_{r}\right)}{C_{d}^{2}}$$

(52) $$L_{3}=\frac{4N_{3}^{2}\emptyset_{3}}{Z}$$

(53) $$M_{3}=\frac{L_{3}}{2}$$

(54) $$t_{3}=\frac{4\left(4L_{3}+4M_{3}\right)}{4R_{3}}$$

where $\emptyset_{3}$ is the magnetic flux of the magnetic circuit of circumferential configuration (see Equation (16)), and $M_{3}$ is the coil mutual inductance of coil of circumferential configuration.

## 3. Results and Discussion

According to the needs of actual production, the mass, damping torque and response time of MR-TVA were taken as development goals in MR-TVA design. The NSGA-II algorithm was used to perform multi-objective optimization on these three configurations, and then the performance of the three optimized configurations was compared.

### 3.1. The Optimization of Mass and Damping Torque

In large-scale construction machinery, some large-scale construction vehicles and rotating machinery that run for a long time in the industry have high output power and monotonic operating conditions during operation. Therefore, when designing the MR-TVA for these application scenarios, the mass and damping torque of MR-TVA should be considered as priority factors.

In this section, the mass and damping torque of MR-TVA is taken as the design goals, and the geometric parameters are taken as the design variables. Due to the inertia ratio of MR-TVA having a certain degree of influence on the amplitude and frequency of the torsional vibration, besides the size constraints, the inertia ratio of MR-TVA is also considered as a constraint condition, and it is limited to a certain empirical range. The effect of the MR-TVA inertia ratio on torsional vibration is shown in Appendix A.

For the three configurations of MR-TVA, the key geometrical parameters were selected as design variables. Based on the relevant dimensions of the used silicone oil shock absorber and the confirmatory finite element simulation, the range of each variable is shown in Table 6, respectively. The constraints of axial single-coil configuration and axial multi-coil configuration are shown in Equation (55), and the constraints of circumferential configuration are shown in Equation (56).

(55) $$\{\begin{array}{l} 48\leq 2C_{t}+R_{t}\leq 58\\ 119\leq R_{n}+I_{r}+C_{g}\leq 129\\ G<20\\ 0.25\leq \frac{I_{d}}{I_{g}}\leq 0.5 \end{array}$$

(56) $$\{\begin{array}{l} 114\leq R_{i}+I_{w}+D_{l}+R_{o}\leq 129\\ G<15\\ 0.25\leq \frac{I_{d}}{I_{g}}\leq 0.5 \end{array}$$

where $I_{d}$ is the moment of inertia of the inertia ring, $I_{g}$ is the sum of the inertia of the MR-TVA housing and the inertia of the crankshaft, and G is the structural mass of the MR-TVA. The typical values for the ratio of the inertia ring moment of inertia to the absorber housing moment of inertia range from 0.25 to 0.5 [18].

**Figure 15:**
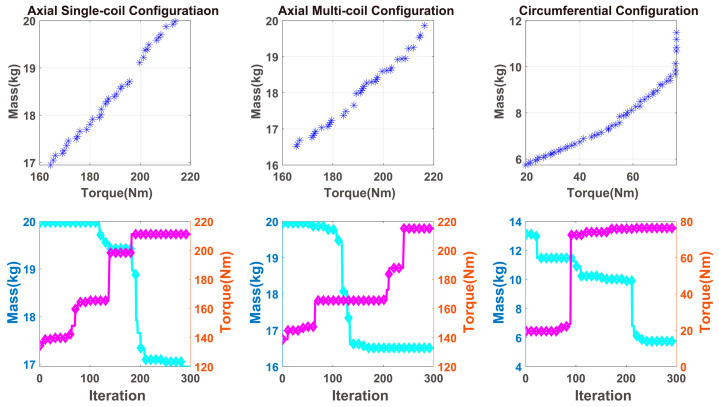
The optimized iteration results of the mass and damping torque of MR-TVA.

The above variables and constraints function were substituted into the magnetically induced damping torque model of three configurations of MR-TVA, and MATLAB was used to perform optimization calculations. The optimization results are shown in Figure 15.

As shown in Figure 15, the blue asterisk in the figure represents the final selectable mass and torque after multiple iterations, while the blue line in the figure represents the iterative process of mass and the red line represents the iterative process of torque. From the iterative process, can known the performance characteristics and upside of the three configurations.

### 3.2. The Optimization of Damping Torque and Response Time

Most common cars and some construction vehicles, such as bulldozers, require a certain output power to operate, and the working conditions change randomly and quickly. The damping torque and response time of MR-TVA should be paid more attention to among the many development goals.

**Figure 16:**
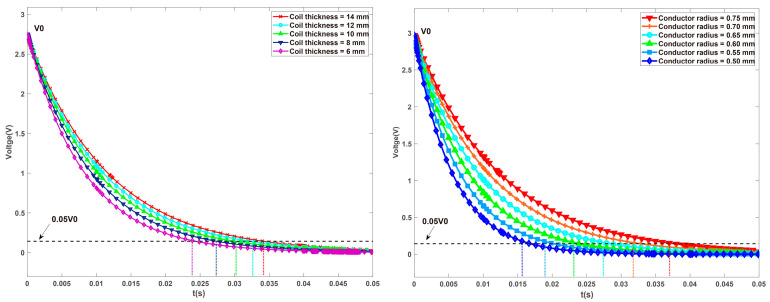
The effect of coil thickness and copper wire diameter on response time.

In this section, the response time and damping torque of MR-TVA are taken as design goals. On the basis of the original design variables, the thickness of the coil and the diameter of the copper wire is considered (according to Figure 14 and the response time model of MR-TVA, the two have a large impact on response time, and the results are shown in Figure 16). The value range of each variable is shown in Table 7. The constraints of axial single-coil configuration and axial multi-coil configuration are shown in Equation (55), and the constraints of circumferential configuration are shown in Equation (56).

The coil thickness ($C_{r}$) and copper wire diameter ($C_{d}$) were selected as design variables, while the other parameters of the coil can be determined by the structure size of the MR-TVA.

**Figure 17:**
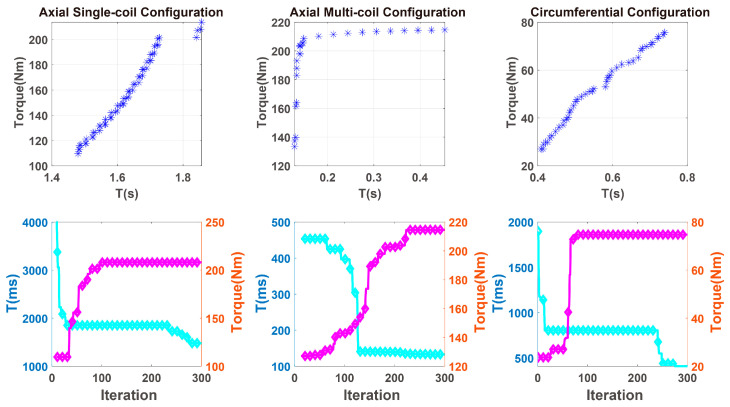
The optimized iteration results of the response time and damping torque of MR-TVA.

The above variables and constraints function were substituted into the damping torque model of three configurations of MR-TVA, and use MATLAB to perform optimization calculations. The optimization results are shown in Figure 17.

As shown in Figure 17, the blue asterisk represents the final selectable response time and torque after multiple iterations, while the blue line in the figure represents the iterative process of response time and the red line represents the iterative process of torque.

Figure 15 and Figure 17 show that when structural parameters change, the target value may not change much; this illustrates a correlation between the structural parameters and shows an intersection of the values of some parameters in the two optimization solution sets. Through the values of these parameters, the optimal values of the three configuration structures can be obtained.

### 3.3. The Optimization Result and Discussion

Based on the MR-TVAs model established above, the three configurations of MR-TVA are optimized twice by the NSGA-II algorithm. The optimal value of each configuration structure parameter is obtained from the intersection of the two optimization results. Three optimal configurations with similar radii and thicknesses are obtained. The performance of the three optimal configurations with the same ampere-turn excitation is shown in Table 8.

The results show that the axial multi-coil configuration provides the largest damping torque (210.01 N.m) and the shortest response time (0.14 s). The damping torque provided by the axial single-coil configuration is close to the axial multi-coil configuration (207.05 N.m), and the response time is the longest (1.84 s). Circumferential configuration exhibits the smallest damping torque (65.60 N.m), but the response time is only 39% of that of the axial single-coil configuration. The mass is only 55% of the axial single-coil configuration.

Among the three configurations, the axial single-coil configuration is relatively simple in structure and magnetic circuit, which makes it convenient for structural design and performance prediction. The axial multi-coil configuration has the highest utilization rate of MR materials. The circumferential configuration has a higher utilization rate of structure than the axial single-coil configuration and axial multi-coil configuration, so the circumferential configuration is lighter than the former two. However, the magnetic circuit of the circumferential configuration is longer, which leads to a lower damping torque of the circumferential configuration. In addition, the axial multi-coil configuration contrast with the axial single-coil configuration, although in the radial fluid gap, there are two magnetic fields of different magnitude and opposite directions, weakening the performance of MR fluids in the radial fluid gap. However, the magnetic field region after superposition is increased in the circumferential direction, and the utilization rate of MR fluid is higher. Therefore, overall, the damping torque of the axial multi-coil configuration is not much different from the axial single-coil configuration and may even be higher under the same excitation. In addition, improving the utilization rate of MR materials will also inhibit the settlement of MR materials to a certain extent.

From the perspective of practical application, the specific selection of the three MR-TVAs needs to be determined according to the engine crankshaft parameters (about the inertia ratio), the working condition characteristics of the engine (determining the response time), and torsional vibration (related to the damping of the absorber). Furthermore, considering all aspects of the engine to design the MR-TVA is the key to the success of the matching design. From the optimization results, the different characteristics of the three configurations can be applied to the different needs of the engine. Moreover, compared with previous similar studies, the MR-TVA provided by this study has a relatively simple structure, is more friendly and convenient for practical applications, has better stability, and is less likely to have problems.

## 4. Conclusions

In this paper, the comprehensive design optimization process of the MR fluid torsional vibration absorber (MR-TVA) was investigated. In the process of design optimization, three structural design schemes of MR-TVA were first proposed, including axial single-coil configuration, axial multi-coil configuration and circumferential configuration. According to Ampere’s loop law and Gauss’s law, the magnetic circuit model of each configuration and the damping torque model of the MR-TVA were established. The finite element model of each configuration was established, and the simulation results of the magnetic field analysis verified the accuracy of the magnetic circuit model.

After obtaining the MR-TVA high-fidelity model, the mass, damping torque and response time of the MR-TVA were taken as the design goals. Two kinds of multi-objective optimizations were performed for each configuration using the NSGA-II algorithm. In the intersection of the two optimization solution set variables, the optimal configuration of the three configurations was determined. After comparing the performance of the three optimal configurations, the following conclusions are obtained:

(1) Axial single-coil configuration can provide a fairly high damping torque, but its response time is much longer than the other configurations. In addition, the axial single-coil configuration has the simplest structure arrangement and is more convenient in design and performance prediction. Therefore, the axial single-coil configuration is suitable for engines and related rotating machines with high output power and relatively simple working conditions.
(2) Axial multi-coil configuration has high utilization of magnetic field and MR material, so it can provide high damping torque. The adjacent arrangement of the coils for axial multi-coil configuration reduces the circuit self-inductance, which makes its response time much less than the other configurations. Therefore, the axial multi-coil configuration is suitable for engines and other rotating machines with high output power and complicated working conditions.
(3) The circumferential configuration has the highest utilization rate of the overall structure and the lowest mass in the MR-TVA of the same specification. However, the circumferential configuration has the longest magnetic circuit and the largest magnetic field loss, which results in the smallest damping torque provided in the MR-TVA of the same specification. Therefore, the circumferential configuration is suitable for engines with low output power and slow speed changes.

From the comparison of optimization results, it can be seen that the three configurations have their own advantages and disadvantages and can be used for torsional vibration reduction under different working conditions. In addition, this paper gives the matching design and optimization method of each configuration corresponding to the actual engine torsional vibration condition and provides a design template for the development of magnetorheological torsional vibration absorbers for different torsional vibration operating conditions.

## Figures and Tables

**Table 1:** Values of fixed parameters of MRF-132DG.

Parameters	Reference Value	Units
Zero field viscosity ($\eta_{0}$)	0.1	Pa.s
Yield viscosity ($\eta_{d}$)	3.8	Pa.s
Zero field shear stress ($\tau_{0}$)	15	Pa
Shear yield stress ($\tau_{\infty }$)	40,000	Pa
Relative permeability ($\mu_{mr}$)	4.5	-

**Table 2:** Values of fixed parameters of MR-TVA.

Configuration	Parameters	Initial Value (mm)
Axial single-coil configuration and Axial multi-coil configuration	Fluid gap (Gap)	1
	Radius of inner ring ($R_{n}$)	50
	Radial size of inertia ring ($I_{r}$)	50
	Thickness of housing ($C_{g}$)	30
	Thickness of cover ($C_{t}$)	10
	Thickness of inertia ring ($R_{t}$)	38
	Thickness of coil ($C_{r}$)	10
	Length of coil ($C_{l}$)	10
	Radius of MR-TVA ($R_{c}$)	130
	Thickness of MR-TVA ($D_{t}$)	60
Circumferential configuration	Radius of inner ring ($R_{i}$)	73
	Thickness of inner ring ($I_{w}$)	12
	Length of winding post ($D_{l}$)	17
	Thickness of outer ring ($R_{o}$)	17
	Opening angle of outer ring ($O_{a}$)	70°
	Thickness of inertia ring ($D_{i}$)	36
	Diameter of winding post ($D_{w}$)	19.6
	Thickness of magnetic permeable ring ($D_{c}$)	5

**Table 3:** The comparison of finite element simulation and modeling calculation results of axial single-coil configuration.

Ampere-Turns (NA)	FEA (mT)	Magnetic Circuit Model (mT)	Error (%)
200	399.57	382.03	4.6
250	499.46	477.54	4.4
300	599.36	573.04	4.4
350	699.25	668.55	4.4
400	799.12	764.06	4.4

**Table 4:** The comparison of finite element simulation and modeling calculation results of axial multi-coil configuration.

Ampere-Turns (NA)	FE Analysis (mT)	Magnetic Circuit Model (mT)	Error (%)
200	[98.68, 408.23]	[102.64, 384.91]	4.0
250	[123.35, 510.28]	[128.30, 481.13]	4.0
300	[148.02, 612.34]	[153.96, 577.36]	3.9
350	[172.69, 714.40]	[179.62, 673.59]	4.0
400	[197.36, 815.89]	[205.28, 769.81]	3.8

**Table 5:** The comparison of finite element simulation and modeling calculation results of circumferential configuration.

Ampere-Turns (NA)	FE Analysis (mT)	Magnetic Circuit Model (mT)	Error (%)
200	68.34	63.09	7.7
250	85.87	78.86	8.2
300	103.04	94.63	8.2
350	120.20	110.40	8.2
400	136.69	126.18	7.7

**Table 6:** Allowable values of the design variables for MR-TVA.

Configuration	Parameters Number	Parameters	Parameters Range (mm)
Axial single-coil configuration & Axial multi-coil configuration	1	$R_{n}$	[10, 60]
	2	$I_{r}$	[30, 60]
	3	$C_{g}$	[15, 50]
	4	$C_{t}$	[5, 20]
	5	$R_{t}$	[40, 50]
Circumferential configuration	1	$R_{i}$	[30, 70]
	2	$I_{w}$	[10, 30]
	3	$D_{l}$	[15, 25]
	4	$R_{o}$	[15, 30]
	5	$O_{a}$(°)	[30, 80]
	6	$D_{i}$	[35, 45]
	7	$D_{w}$	[15, 30]
	8	$D_{c}$	[5, 15]

**Table 7:** Allowable values of the design variables for MR-TVA.

Configuration	Parameters Number	Parameters	Parameters Range (mm)
Axial single-coil configuration & Axial multi-coil configuration	1	$R_{n}$	[10, 60]
	2	$I_{r}$	[30, 60]
	3	$C_{g}$	[15, 50]
	4	$C_{t}$	[5, 20]
	5	$R_{t}$	[40, 50]
	6	$C_{r}$	[5, 15]
	7	$C_{d}$	[0.8, 1.2]
Circumferential configuration	1	$R_{i}$	[30, 70]
	2	$I_{w}$	[10, 30]
	3	$D_{l}$	[15, 25]
	4	$R_{o}$	[15, 30]
	5	$O_{a}$(°)	[30, 80]
	6	$D_{i}$	[35, 45]
	7	$D_{w}$	[15, 30]
	8	$D_{c}$	[5, 15]
	9	$C_{r}$	[5, 10]
	10	$C_{d}$	[0.5, 1.2]

**Table 8:** Optimization results and comparison of MR-TVA.

Parameter	Axial Single-Coil Configuration		Axial Multi-Coil Configuration		Circumferential Configuration	
Optimal Values (mm)	$R_{i}$	60	$R_{i}$	60	$R_{i}$	64
	$I_{r}$	45	$I_{r}$	43	$I_{w}$	10
	$C_{g}$	23	$C_{g}$	23	$D_{l}$	15
	$C_{t}$	6	$C_{t}$	6	$R_{o}$	27
	$R_{t}$	40	$R_{t}$	42	$O_{a}$°	78
	$C_{r}$	5	$C_{r}$	5	$D_{i}$	43
	$C_{d}$	1.2	$C_{d}$	1.0	$D_{w}$	29
					$D_{h}$	7
					$C_{r}$	5
					$C_{d}$	1.2
Radius (mm)	129		127		129	
Mass (kg)	19.59		19.23		11.03	
Response (s)	1.84		0.14		0.45	
Torque (N.m)	207.05		210.01		65.60	

**Figure A1:**
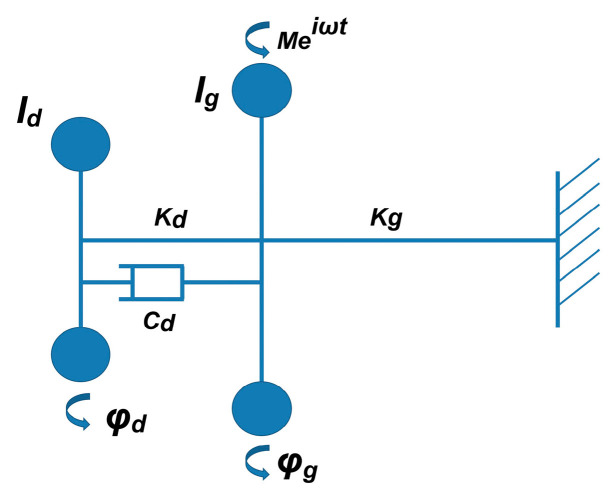
Dual degrees of freedom torsional system.

**Figure A2:**
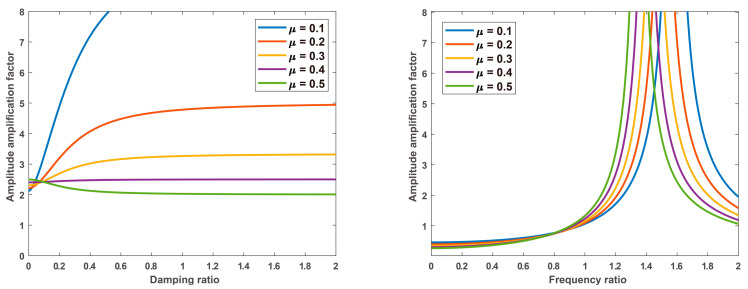
The influence of the inertia ratio μ on amplitude and frequency.

## Data Availability

Not applicable.

## Appendix A

When analyzing the shafting multi-degree-of-freedom torsion system, the equivalent of the shafting multi-degree-of-freedom system is often simplified to a single torsional pendulum system. After installing the MR-TVA on the free end of the crankshaft, the shaft system and the MR-TVA can be simplified into a dual degrees of freedom torsional system. The torsional system is shown in Figure A1.

According to D’Alembert’s principle combined with the equivalent model, the response of the system in Figure A1 can be mathematically expressed as:

(A1) $$\{\begin{array}{l} I_{d}{\ddot{\varphi }}_{d}+C_{d}\left({\dot{\varphi }}_{d}-{\dot{\varphi }}_{g}\right)+K_{d}\left(\varphi_{d}-\varphi_{g}\right)=0\\ I_{g}{\ddot{\varphi }}_{g}+C_{d}\left({\dot{\varphi }}_{g}-{\dot{\varphi }}_{d}\right)+K_{d}\left(\varphi_{g}-\varphi_{d}\right)+K_{g}\varphi_{g}=Me^{i\omega t} \end{array}$$

where $I_{d}$ and $I_{g}$ are the equivalent inertia of the inertial body of the torsional vibration absorber and the equivalent inertia of the shaft system; $K_{d}$ and $K_{g}$ are the torsional stiffness of the torsional vibration absorber and the equivalent stiffness of the shaft system, respectively; $C_{d}$ is the damping coefficient of the torsional vibration absorber; $\varphi_{d}$ and $\varphi_{g}$ are the torsion angle of the inertial body of the torsional vibration absorber and the torsion angle of the shaft system, respectively; and $Me^{i\omega t}$ is the equivalent excitation torque of the shaft system.

After the MR-TVA is installed in the crankshaft system, the absorber housing is fixed with the free end of the crankshaft, and the inertia ring and the absorber housing are connected by the friction torque provided by the fluid medium, so the inertia ring and the housing can be regarded as a double mass for the torsional pendulum. Through the solution analysis of its differential equation of motion, it can be seen that the influence of the inertia ratio μ ($I_{d}/I_{g}$) on the torsional vibration amplitude and torsional vibration frequency is shown in Figure A2:

As shown in Figure A2, when the damping ratio is large, the torsional amplitude of the shafting system will decrease with the increase of the inertia ratio. In addition, the natural frequency of the shafting system will decrease with the increase of the inertia ratio.

The inertia ratio of the MR-TVA has a quite influence on the amplitude amplification factor and also has a certain degree of influence on the frequency shift of the system. Therefore, the inertia ratio of the MR-TVA should also be considered when designing the magnetic circuit structure of the MR-TVA and subsequent optimization.
